# Clinicopathological and prognostic features of hepatitis B virus-associated diffuse large B-cell lymphoma: a single-center retrospective study in China

**DOI:** 10.1186/s13027-021-00396-x

**Published:** 2021-08-17

**Authors:** Dao-guang Chen, Gang Chen, Chang Wang, Long-feng Ke, Hui Wu, Hong-ming He, Yu Yang, Yan-ping Chen

**Affiliations:** 1 Department of Head-neck Tumor & Lymphoma, Fujian Medical University Cancer Hospital and Fujian Cancer Hospital, Fuzhou, 350014 Fujian Province China; 2 Department of Pathology, Fujian Medical University Cancer Hospital and Fujian Cancer Hospital, No 420, Fuma Road, Fuzhou, 350014 Fujian Province China; 3 Fujian Provincial Key Laboratory of Tumor Biotherapy, Fuzhou, 350014 China; 4 Department of Molecular Pathology, Fujian Medical University Cancer Hospital and Fujian Cancer Hospital, Fuzhou, 350014 China

**Keywords:** Prognosis, Hepatitis B virus, Diffuse large B‑cell lymphoma, R-CHOP

## Abstract

**Background:**

While the epidemiologic association between hepatitis B virus (HBV) infection and diffuse large B-cell lymphoma (DLBCL) is established, little is known about the pathological characteristics and outcome of DLBCL arising in patients with HBV infection.

**Methods:**

We retrospectively studied a cohort of 420 patients with DLBCL for the incidence of HBV infection, and the clinicopathologic features and prognostic factors in HBsAg-positive DLBCL patients in China, a hepatitis B endemic area.

**Results:**

In our study, 127 (30.2%) patients were HBsAg-positive. HBsAg-positive DLBCL displayed a younger median onset age (50 vs. 54 years, *P* = 0.002), more frequent involvement of the spleen (19.7% vs. 6.1%, *P* < 0.001), less frequent involvement of the small and large intestine (2.3% vs. 11.2%, *P* = 0.003), more advanced disease (stage III/IV: 56.7% vs. 45.1%, *P* = 0.028), and lower expression rate of MYC (49.1% vs. 66.7%, *P* = 0.026). The median follow-up time was 61.9 months. Univariate analysis showed that there was no significant difference in overall survival (OS) between HBsAg-negative and -positive DLBCL (*P* = 0.577). In the HBsAg-positive DLBCL subgroup, age older than 60 years, advanced disease, elevated lactate dehydrogenase (LDH), spleen involvement, B symptoms (fever, night sweats, weight loss), and double expressers of MYC and BCL2 had a significantly worse outcome, and patients treated with R-CHOP (rituximab plus cyclophosphamide, doxorubicin, vincristine, and prednisone) had a better prognosis. Multivariate analysis further confirmed that spleen involvement and rituximab use were independent prognostic factors in HBsAg-positive DLBCL patients.

**Conclusions:**

Our study indicates that HBsAg-positive DLBCL has unique clinicopathological features and independent prognostic factors. Moreover, under antiviral prophylaxis, the survival of DLBCL patients with HBV infections was comparable to that of HBV-negative patients, and the use of rituximab significantly improved OS in HBsAg-positive DLBCL patients.

**Supplementary Information:**

The online version contains supplementary material available at 10.1186/s13027-021-00396-x.

## Introduction

Diffuse large B-cell lymphoma (DLBCL), the most frequent type of B-cell lymphoma, constitutes 25–35% of adult non-Hodgkin lymphoma cases in developed countries and a higher percentage in developing countries [1, 2]. This entity encompasses distinct morphological, molecular, and clinicopathological subgroups. At present, CHOP/CHOP-like (cyclophosphamide, doxorubicin, vincristine, and prednisolone) or R-CHOP (CHOP plus rituximab) regimens are the standard first-line therapy for DLBCL patients [3–5]. However, there are certain disadvantages to first-line chemotherapy, including hepatitis B virus (HBV) reactivation in response to MabThera and activated or aggravated HBV infection status [6–8]. Therefore, the prophylaxis and management of this complication has become a major issue in the management of DLBCL patients with chronic HBV infection. The data indicate that HBsAg positivity is an unfavorable prognostic factor for patients treated with MabThera [9–11], but whether a patient’s prognosis is influenced by the presence of an HBV infection has been little reported.

At present, HBV infection remains a significant public health problem. Over 2 billion people have evidence of HBV infection, and approximately 350 million are chronic carriers worldwide [12]. China is an endemic area for HBV infection with a HBsAg positivity rate of 7.18% in the general population and around 93 million chronically infected patients [13]. HBV is a hepatotrophic and lymphotropic virus, and HBV infection and replication in lymphocytes might contribute to the development of lymphoproliferative disorders [14]. Many studies have found a higher prevalence of HBV infection in non-Hodgkin’s lymphoma (NHL) patients, especially those with DLBCL, than in the general population [15–23]. While the epidemiological association between HBV infection and DLBCL is established, few articles have systematically investigated the clinicopathological characteristics and adverse prognostic factors of DLBCL patients with HBV infection. Some researchers reported that the survival of DLBCL patients with HBV infection is poor compared with DLBCL patients without HBV infection [24–29], while others found that, in both HBsAg-positive and -negative DLBCL patients, the median overall survival (OS) is similar [30, 31].

Because HBV infection is very common in China, it is important to understand its impact on clinicopathological characteristics and outcomes among DLBCL patients receiving treatment. Therefore, the main aim of our study was to retrospectively investigate the clinicopathological characteristics and prognosis of DLBCL patients with HBV infection and compare them with those of patients without HBV infection in a cohort of 420 patients with DLBCL.

## Material and methods

### Patients and tissue collection

This retrospective study was performed on 420 patients with DLBCL who were originally diagnosed and treated at Fujian Cancer Hospital, China, from March 2011 to February 2018. Cases of secondary DLBCL transformed from low grade B-cell lymphoma, de novo CD5-positive DLBCL, and specific entities (such as EBV-positive diffuse large B-cell lymphoma, NOS) were excluded from the selection. All cases were reviewed by two hematopathologists to confirm the histological diagnosis according to the criteria of the 2017 World Health Organization (WHO) classification of tumors of hematopoietic and lymphoid tissues [2]. The staging was determined according to the Ann Arbor Staging Criteria. Response to treatment was evaluated according to the standard response criteria (physical examination, CT scan, and bone marrow biopsy), and imaging studies were performed after every two cycles of chemotherapy. All patients received a standard CHOP (cyclophosphamide, doxorubicin, vincristine, and prednisone) regimen or rituximab plus CHOP (R-CHOP) as the first-line treatment. After chemotherapy, patients with residual disease or bulky disease were routinely given regional radiation therapy (RT) to the involved sites. HBsAg-positive patients routinely received lamivudine or entecavir prophylactic treatment, starting from the beginning of chemotherapy and stopping one year after its completion. When the HBV deoxyribonucleic acid (DNA) in the serum of HBsAg-positive patients was below 2 × 10^3^ copies/ml, rituximab was commenced. Clinical, laboratory, and follow-up data were obtained from patient medical records and charts. This study was carried out in accordance with the Declaration of Helsinki and written informed consent was obtained from the patients or their legal guardians. The study protocol was approved by the institutional review boards of Fujian Cancer Hospital.

### Histology and immunohistochemical analysis

Biopsy specimens were fixed in 10% formalin and embedded in paraffin after routine histological tissue processing. Three to four micrometer-thick formalin-fixed paraffin-embedded tissue (FFPE) sections were stained with hematoxylin–eosin (HE) for microscopic examination. All immunohistochemistry assays were performed on diagnosed patient tissues available in the form of FFPE tissue blocks using Ventana-Benchmark XT automated instruments at the Department of Pathology. Immunostaining on paraffin sections was performed for CD20, CD21, CD3, Ki-67, CD10, BCL-6, IRF4/MUM-1, BCL-2, MYC, cyclin D1, CD5, CD30, and p53 (all clones from Maixin Biotech Co., Ltd., Fuzhou, China). Conditions for individual immunohistochemistry assays including antigen retrieval and antibody dilutions varied according to the antibody used and were determined by standard optimization and validation procedures. Positive and negative staining controls were included as appropriate. CD10, BCL6, and IRF4/MUM1 were each considered positive if ≥ 30% of the tumor cells were positively stained [2]. According to the 2016 revised WHO guidelines [2], BCL2 was considered positive if ≥ 50% of the tumor cells were positive, and MYC was considered positive if ≥ 40% of the tumor cell nuclei were positive. To define elevated p53 protein expression, a cut-off was set at 50% positively staining cells as proposed by Xu-Monette and colleagues [32].

### HBV detection

Routine screening for viral markers including HBsAg, hepatitis-B surface antibody (HBsAb), hepatitis-B e antigen (HBeAg), hepatitis-B e antibody (HBeAb), and hepatitis-B core antibody (HBcAb) was performed by chemiluminescence immunoassay on an Architect-i2000 instrument (Abbott Laboratories). Real-time quantitative polymerase chain reaction was used to determine the HBV DNA copy number before chemotherapy if HBsAg was positive.

### In situ hybridisation

Epstein-Barr virus (EBV) detection by in situ hybridization was performed in all cases using FITC-labeled oligonucleotide probe supplied by Ventana to detect EBV-encoded RNA (EBER). In situ hybridization for EBER was conducted on FFPE sections on an automated stainer (Ventana-Benchmark XT) according to the manufacturer's instructions. The visualization was achieved using the ISH iView system with alkaline phosphatase and NT/BCIP substrate, with fast red as a contrast. EBV-positive nasopharyngeal carcinoma was chosen as the positive control.

### Statistical analysis

SPSS software (version 20.0, IBM, Armonk, NY, USA) was used for all statistical analyses. The patient’s characteristics were compared across different subgroups using Pearson’s chi-squared test or Fisher’s exact test. OS was determined from the date of diagnosis to the date of death or the last follow-up. Univariate analysis and survival curves were generated by the Kaplan–Meier method and compared using the log-rank test. Multivariate analysis was conducted with the Cox proportional hazards model, which included the variables that were significant in the univariate analysis. A *P* value of < 0.05 was considered statistically significant.

## Results

### Baseline clinicopathological characteristics in all patients with DLBCL

The study group included 237 males and 183 females with a male-to-female ratio of 1.3:1. The age of onset ranged from 16 to 86 years, with a median age of 53 years and an average age of 51.6 years. Among these patients, 119 patients (28.3%) were older than 60 years. There were 66 (15.7%), 150 (35.7%), 78 (18.6%), and 126 (30.0%) patients with Ann Arbor stage I, II, III, and IV disease, respectively. There were 335 (79.8%) and 85 (20.2%) patients with low International Prognostic Index (IPI) and high IPI, respectively. B symptoms, bulky mass (≥ 7.5 cm), elevated lactate dehydrogenase (LDH), and extra-nodal sites ≥ 2 were identified in 43 (10.2%), 74 (17.6%), 194 (46.2%), and 71 (16.9%) patients, respectively. The follow-up time ranged from 58.0 to 65.8 months (median, 61.9 months). One hundred and forty-four of the 420 patients died of the disease. The 3- and 5-year OS rates were 71.3% and 64.9%, respectively.

### The immunophenotype of all patients with DLBCL

**Fig. 1:**
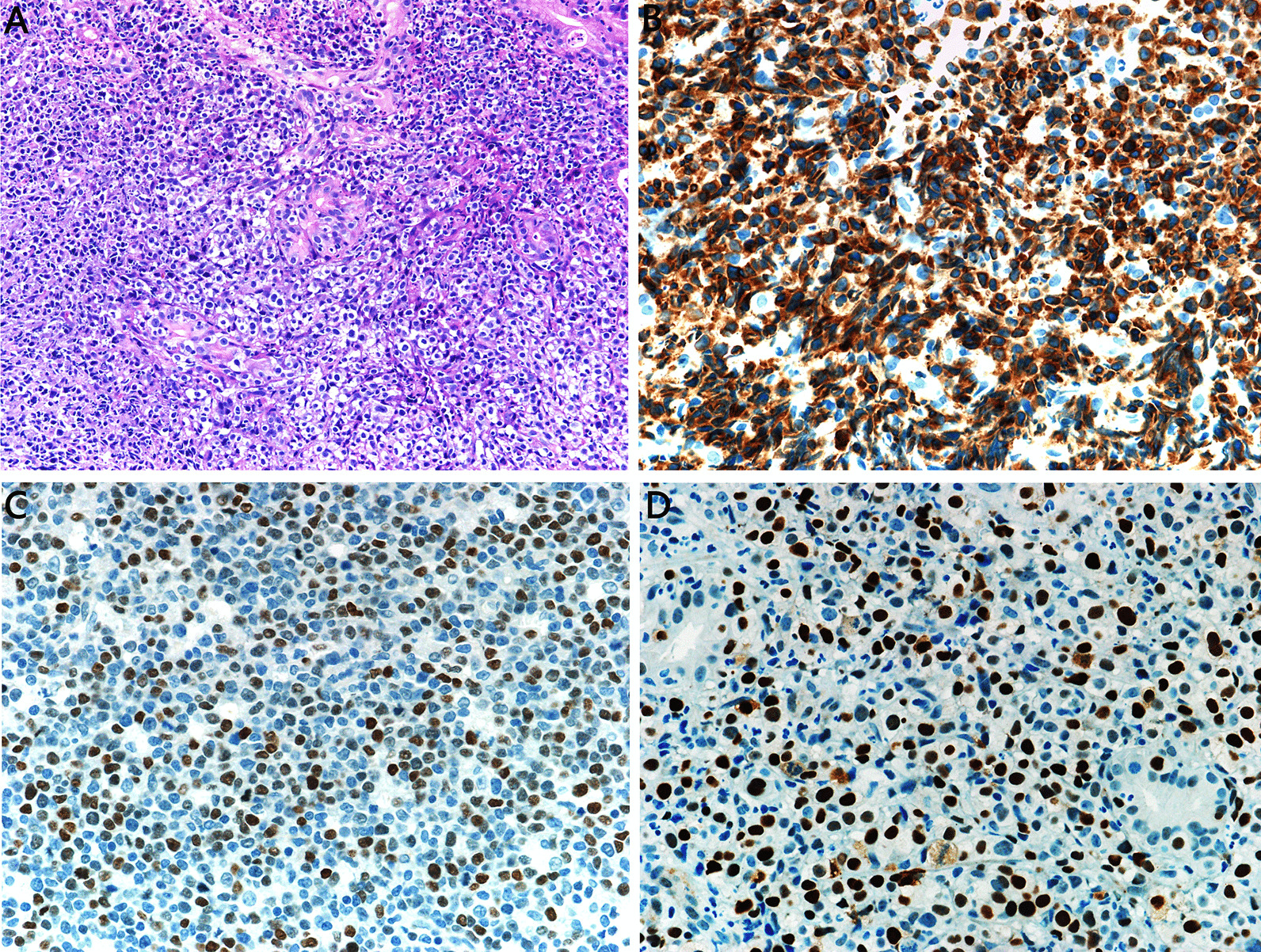
Immunohistochemical staining of diffuse large B-cell lymphoma (DLBCL) tissues. **A** Hematoxylin and eosin staining of DLBCL (× 400). **B** BCL-2 was positively expressed in the nuclei of DLBCL cells (× 400). **C** c-MYC was positively expressed in the nuclei of DLBCL cells (× 400). **D** p53 was positively expressed in the nuclei of DLBCL cells (× 400)

All cases were strongly positive for CD20 and negative for CD3, CD5, and cyclin-D1. The germinal center B-cell (GCB) and non-GCB subtypes of DLBCL were classified based on immunohistochemical staining of CD10, BCL-6, and MUM-1 by Hans' algorithm. Among 420 cases, 266 (63.3%) cases demonstrated a non-GCB immunophenotype, whereas 154 (36.7%) cases demonstrated a GCB immunophenotype. The ratio of non-GCB to GCB phenotype was 1.7:1. The Ki-67 proliferation index was generally high, and was usually much more than 40%, with > 60% in 331 (78.8%) cases. There were 271/360 (75.3%) and 106/174 (60.9%) cases that tested positive for BCL-2 and MYC, respectively, and 79/172 (45.9%) cases were positive for both BCL2 and MYC (Fig. 1A–C). Coexpression of these two proteins (so-called double expressers) was more frequent in the non-GCB subtype. There were 27/114 (23.7%) cases that tested positive for p53 (Fig. 1D). CD30 was weakly and focally expressed in some cases. All cases were negative for EBER.

### Clinicopathological features of HBsAg-positive group compared with HBsAg-negative group

**Fig. 2:**
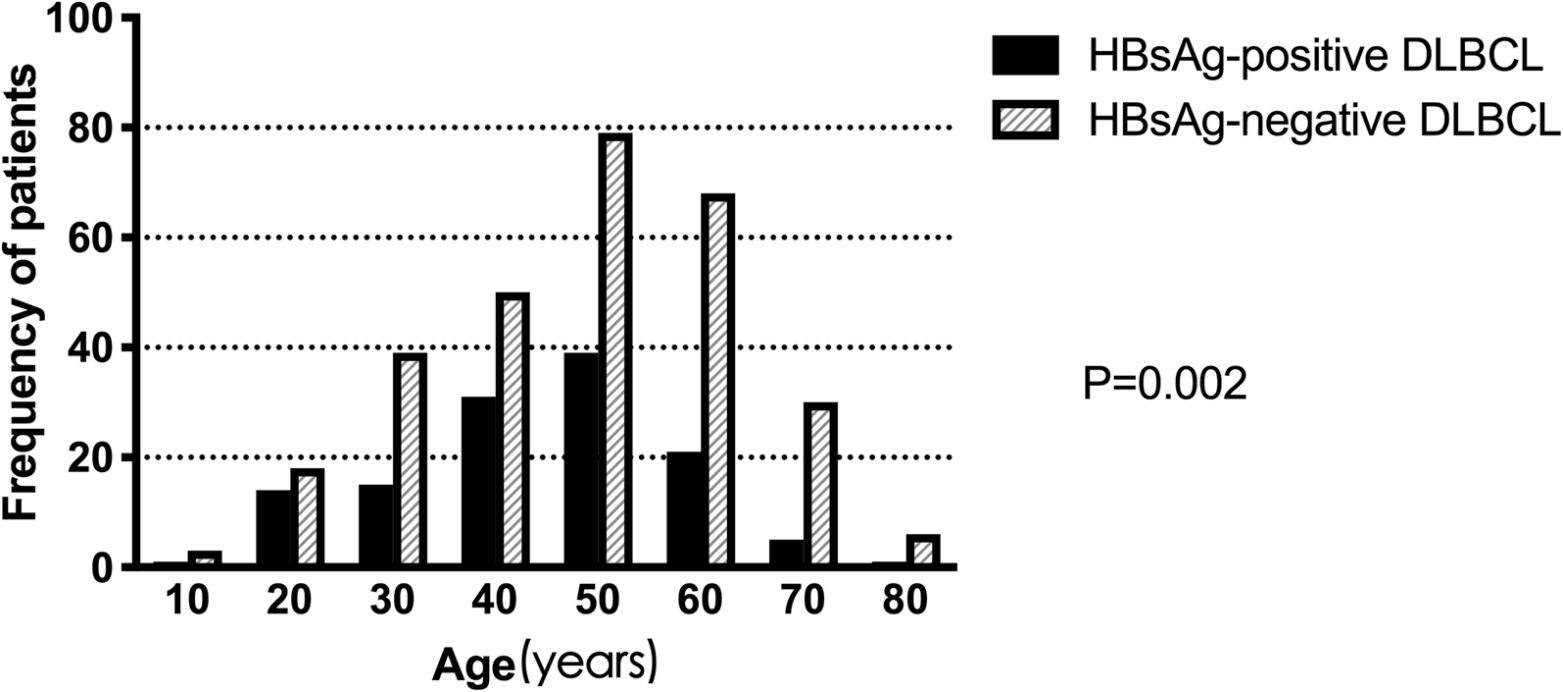
Age distribution of diffuse large B-cell lymphoma patients in HBsAg-positive and -negative groups

The clinicopathological characteristics of HBsAg-positive and -negative patients are shown in Tables 1, 2. One hundred and twenty-seven patients were HBsAg-positive (127/420, 30.2%) and 293 (69.8%) were HBsAg-negative. Compared with patients in the HBsAg-negative group, patients in the HBsAg-positive group were of a younger age with a median onset age of 50 years (range 18–82) versus 54 years (range 16–86) in the HBsAg-negative group (*P* = 0.002). In the HBsAg-positive group, fewer patients were over 60 years (17.3% vs. 33.1%, *P* < 0.001, Fig. 2). Furthermore, DLBCL in the HBsAg-positive group showed more frequent involvement of the spleen (19.7% vs. 6.1%, *P* < 0.001), more advanced disease (stage III/IV: 56.7% vs. 45.1%, *P* = 0.028), less frequent involvement of the small and large intestine (2.3% vs. 11.2%, *P* = 0.003), and lower expression rate of MYC (49.1% vs. 66.7%, *P* = 0.026). Eighty cases (80/127, 63.0%) in the HBsAg-positive group compared with 186 (186/293, 63.4%) in the HBsAg-negative group were classified as being of the non-GCB subtype of DLBCL. There was no significant difference in the percentage of non-GCB patients between these two groups (*P* = 0.924). Furthermore, there was no significant difference in sex, bulky mass, performance status, B symptoms, elevated LDH, extra-nodal sites ≥ 2, high IPI, use of rituximab, Ki-67 proliferation index, expression of BCL2, p53 expression, and coexpression of BCL2 and MYC between the HBsAg-positive and -negative groups.

**Table 1:** Clinical characteristics of HBsAg-positive and -negative patients with diffuse large B-cell lymphoma

Factors	HBsAg-positive patients (n = 127)		HBsAg-negative patients (n = 293)		*P* value
	No	%	No	%	
Age > 60	22	17.3	97	33.1	0.001*
Gender					0.116*
Male	79	62.2	158	53.9	
Female	48	37.8	135	46.1	
Bulky mass (≥ 7.5 cm)	22	17.3	52	17.7	0.916*
Special sites involvement					
Liver	6	4.7	10	3.4	0.519*
Spleen	28	22.0	19	6.5	< .001*
Stomach	11	8.6	34	11.6	0.371*
Intestinal	3	2.3	33	11.2	0.003*
Waldeyer’ string	26	20.5	71	24.2	0.401*
Bone	9	7.1	12	4.1	0.196*
Performance status					0.425*
0–1	115	90.6	272	92.8	
2–4	12	9.4	21	7.2	
B symptoms present	28	22.0	76	26.0	0.396*
Elevated LDH	59	46.5	135	46.1	0.943*
Ann Arbor stage					0.028*
I–II	55	43.3	161	54.9	
III–IV	72	56.7	132	45.1	
Extra-nodal sites ≥ 2	22	17.3	49	16.7	0.880*
IPI score					0.653*
0–1	103	81.1	232	79.2	
3–5	24	18.9	61	20.8	
Use of rituximab	60	47.2	143	48.8	0.769*
Response to chemotherapy					0.300^†^
Complete response	78	61.4	196	66.9	
Partial response	40	31.5	83	28.3	
Stable disease	1	0.8	4	1.4	
Progressive disease	7	5.5	10	3.4	
Not available	1	0.8	0	0.0	

*IPI* International Prognostic Index, *LDH* lactate dehydrogenase

*χ2 test for independence in HBsAg-positive compared with HBsAg-negative patients

^†^Fisher exact test for independence in HBsAg-positive compared with HBsAg-negative patients

**Table 2:** Prognostic factor of HBsAg-positive and -negative patients with diffuse large B-cell lymphoma

Prognostic factor	HBsAg-positive patients		HBsAg-negative patients		*P* value
	n/N	%	n/N	%	
Cell of origin (non-GCB)	80/127	63.0	186/293	63.4	0.924
Ki-67 ≥ 80%+	79/127	62.2	192/293	65.5	0.513
Bcl-2+	89/113	78.8	182/247	73.7	0.300
MYC+	28/57	49.1	78/117	66.7	0.026
Both MYC and Bcl-2+	22/56	39.2	57/116	45.2	0.224
P53+	6/35	17.1	21/79	26.6	0.274

### Treatment responses of HBsAg-positive group compared with HBsAg-negative group

There was no significant difference in the treatment response in patients with R-CHOP/CHOP regimen between HBsAg-positive and -negative groups (Additional file 1: Tables S1, S2). Furthermore, in the HBsAg-positive DLBCL group, though there was no significant statistical difference in complete response between the R-CHOP and CHOP groups, complete response in the R-CHOP group was better than that in the CHOP group with a higher rate of complete response (70.0% vs. 54.5%, *P* = 0.082, Table 3). Additionally, for HBsAg-positive DLBCL patients, remission rate did not differ between the GCB and non-GCB subtypes (*P* = 0.252, Table 4).

**Table 3:** Responses to treatment by HBsAg-positive and -negative DLBCL patients according to R-CHOP and CHOP regimens

Treatment response	HBsAg-positive patients			HBsAg-negative patients		
	R-CHOP (n = 60)	CHOP (n = 66)	*P* value	R-CHOP (n = 143)	CHOP (n = 150)	*P* value
CR/CRu	42	36	0.082	114	82	0.001
PR	15	25		28	55	
SD	0	1		1	3	
PD	3	4		0	10	

*CHOP* cyclophosphamide, doxorubicin, vincristine and prednisone, *R-CHOP* addition of rituximab to CHOP, *CR* complete response, *PR* partial response, *SD* stable disease, *PD* progressive disease

**Table 4:** Treatment response between GCB subgroup and non-GCB subgroups in HBsAg-positive and -negative DLBCL patients

Treatment response	HBsAg-positive patients			HBsAg-negative patients		
	GCB (n = 47)	non-GCB (n = 79)	P-Value	GCB (n = 107)	non-GCB (n = 186)	*P* value
CR/CRu	42	46	0.252	73	123	0.718
PR	15	27		29	54	
SD	0	1		2	2	
PD	3	5		3	7	

*CR* complete response, *PR* partial response, *SD* stable disease, *PD* progressive disease

### Univariate survival analysis between HBsAg-positive and negative group

**Fig. 3:**
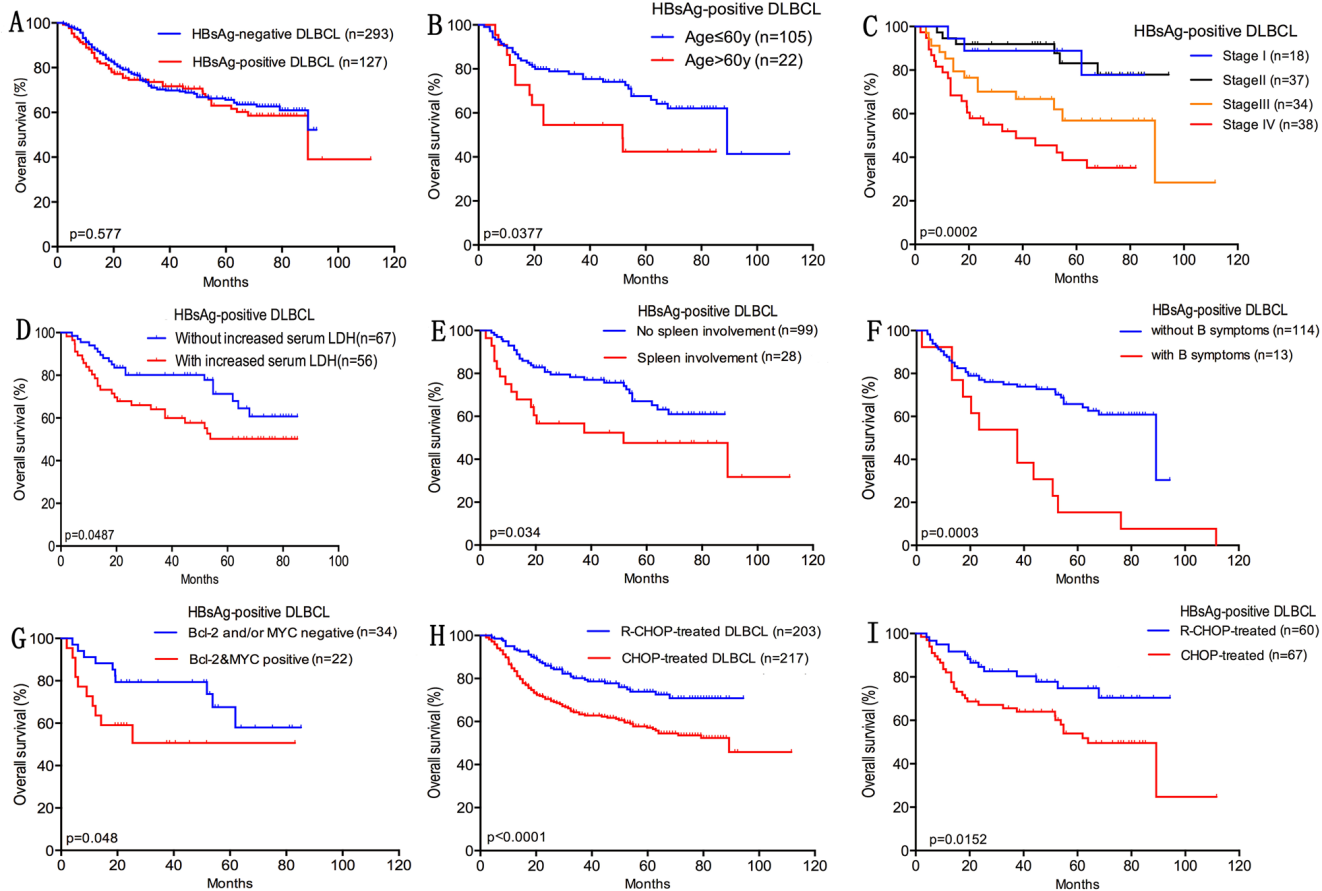
Survival analysis of HBsAg-positive diffuse large B-cell lymphoma patients. **A** Overall survival (OS) of HBsAg-positive and -negative DLBCL patients. **B** OS of HBsAg-positive DLBCL patients of more than 60 years old and less than 60 years old. **C** OS of clinical staging in HBsAg-positive DLBCL patients. **D** OS of HBsAg-positive DLBCL patients with and without elevated LDH. **E** OS of HBsAg-positive DLBCL patients with and without spleen involvement. **F** OS of HBsAg-positive DLBCL patients with and without B symptoms. **G** OS of HBsAg-positive DLBCL patients with and without coexpression of BCL2 and MYC. **H** OS of DLBCL patients treated with R-CHOP or CHOP regimens. **I** OS of HBsAg-positive DLBCL patients treated with R-CHOP or CHOP regimens

The median follow-up time was 66.9 months (range 60.7–73.1) for the HBsAg-positive group and 63.9 (range 60.1–67.7) months for the HBsAg-negative group. Patients in the HBsAg-positive group did not show significantly worse OS than those in the HBsAg-negative group with a 3-year OS of 73.6% versus 70.2% and a 5-year OS of 63.0% versus 65.6% (*P* = 0.577, Fig. 3A). However, survival analysis of different age groups showed that there was a tendency that HBV infection affected the prognosis of DLBCL patients under 40 years of age (Additional file 1: Figure S1, *P* = 0.094). There was a better outcome regarding OS in patients under 60 years of age in the HBsAg-positive group (P = 0.0377, Fig. 3B). In the HBsAg-positive DLBCL subgroup, patients with advanced disease, elevated LDH, spleen involvement, or B symptoms had a significantly worse outcome (*P* = 0.0002, *P* = 0.0487, *P* = 0.034, and *P* = 0.0003, respectively, Fig. 3C–F). One hundred and twenty of the 127 HBsAg-positive patients had HBeAg and HBV DNA loads. For the 120 HBsAg-positive DLBCL patients, the survival analysis showed that OS did not differ between HBV DNA- and HBeAg-positive and HBV DNA- and HBeAg-negative patients (*P* = 0.536 and *P* = 0.534, respectively). In the HBsAg-positive DLBCL patients, coexpression of BCL2 and MYC had a significantly worse outcome (*P* = 0.048, Fig. 3G). However, high expression of Ki-67 proliferation index, BCL2, MYC, and p53 did not affect prognosis (*P* = 0.676, *P* = 0.074, *P* = 0.167, and *P* = 0.875, respectively). For HBsAg-positive DLBCL patients, OS did not differ between the GCB and non-GCB subtypes (*P* = 0.124). In our study, 203 of the 420 (203/420, 48.1%) patients were treated with R-CHOP and the remainder were treated with CHOP. The survival analysis revealed that patients treated with R-CHOP had a better prognosis (*P* < 0.001, Fig. 3H). Correspondingly, there was better OS in the HBsAg-positive DLBCL patients treated with R-CHOP compared with those that received CHOP (*P* = 0.0152, Fig. 3I). The poor prognostic factors between HBsAg-positive and -negative groups were similar except B symptoms (Table 5 and Additional file 1: Figure S2).

**Table 5:** Univariate analyses in HBsAg-positive and -negative DLBCL patients

Characteristics	HBsAg-positive DLBCL patients		HBsAg- negative DLBCL patients	
	N (total = 127)	Log-rank P-value	N (total = 293)	Log-rank *P* value
Sex				
Male	79	0.5748	158	0.6699
Female	48		135	
Age				
> 60	22	0.0377	97	< 0.0001
≤ 60	105		196	
Ann Arbor stage				
I	18	0.0002	48	< 0.0001
II	37		113	
III	34		44	
IV	38		88	
B symptoms				
Present	28	0.0003	76	0.697
Absent	99		217	
Elevated LDH				
Present	67	0.0487	135	< 0.0001
Absent	56		158	
Unknown	4		–	
Treatment with rituximab				
Yes	60	0.0152	143	0.002
No	67		150	
Spleen involvement				
Yes	28	0.034	19	0.002
No	99		274	
Bcl-2&Myc positive				
Yes	34	0.048	57	0.037
No	22		59	
Unknown	71		177	
Cell of origin				
GCB	47	0.124	107	0.103
Non-GCB	80		186	
HBV DNA copies				
< 1000 copies/ml	51	0.536	–	–
> 1000 copies/ml	69			
Unknown	7			
HBeAg				
Positive	25	0.534	–	–
Negative	95			
Unknown	7			

### Multivariable survival analysis between HBsAg-positive and negative group

Multivariate analysis further confirmed that spleen involvement and use of rituximab were independent prognostic factors in HBsAg-positive DLBCL patients (HR 3.725, 95% CI 1.444–9.614, *P* = 0.007, and HR 0.294, 95% CI 0.102–0.843, *P* = 0.023, respectively, Table 6), while clinical staging and use of rituximab were independent prognostic factors in HBsAg-negative DLBCL patients (Additional file 1: Table S3).

**Table 6:** Multivariate Cox proportional hazards model for analysis of survival in HBsAg-positive DLBCL patients

Factors	HR	95% CI		*P* value
		Lower	Upper	
Age/year				
> 60	1.940	0.528	7.132	0.319
≤ 60	1	-	-	
Ann Arbor stage				
I–II	1	–	–	0.532
III–IV	1.500	0.421	5.337	
B-symtoms				
Yes	1.969	0.565	6.860	0.287
No	1	–	–	
Elevated LDH				
Yes	1.345	0.448	4.037	0.598
No	1	–	–	
Treated with rituximab				
Yes	0.294	0.102	0.843	0.023
No	1	–	–	
Spleen involvement				
Yes	3.725	1.444	9.614	0.007
No	1	–	-	
Bcl-2&Myc Positive				
Yes	2.270	0.879	5.862	0.090
No	1	–	–	

## Discussion

HBV infection is endemic in some parts of Asia, Africa, and South America, and remains a significant public health problem in these areas. To our knowledge, this is one of the few studies to examine whether the presence of HBV infection affected the clinical characteristics and outcomes in patients with DLBCL, our study included 127 HBsAg-positive patients, which was one of the largest number of patients in all studies of this disease. In our study, HBV prevalence in DLBCL patients was approximately 30.2%, which was similar or higher than the 13.3%–30.9% observed in previous studies from China, Taiwan, Singapore, and another Asian country [9, 11, 24, 29, 31, 33–35]. These results suggested that in HBV-prevalent regions, a certain percentage of DLBCL patients are chronically infected with HBV.

At present, many epidemiological studies, case–control studies, and meta-analyses have shown that HBV infection may increase the incidence of several types of B-cell lymphoma, especially DLBCL. While the epidemiological association between HBV infection and DLBCL is established, the etiopathological role of HBV in lymphomagenesis remains largely unknown. Ren and colleagues [37] performed the first comprehensive genomic and transcriptomic study on DLBCL samples from patients with HBV infection and identified distinct molecular features of these tumors, suggesting a direct link between HBV infection and B-cell malignancy. They found an enhanced rate of mutagenesis and a distinct set of mutational targets in HBV-associated DLBCL genomes, which could be partially explained by the activities of APOBEC and activation-induced cytidine deaminase. Moreover, the HBV-associated gene expression signature is impacted by the enrichment of genes regulated by BCL6, FOXO1, and ZFP36L1. They also showed that an antigen-independent mechanism is favored in HBV-related lymphomagenesis.

In our study, we further found HBsAg-positive DLBCL patients showed unique clinical features, including an earlier disease onset age, much more common involvement of the spleen, less frequent involvement of the small and large intestine, and more advanced disease. An earlier disease onset age in HBsAg-positive patients was reported in studies conducted in Korea [] and China [11, 24, 29, 33, 36]. More advanced stage and frequent involvement of the spleen in HBsAg-positive DLBCL patients were also reported by some groups [24, 29, 36], but not by others [9, 11, 37]. This discrepancy might be ascribed to the small sample sizes [37] and different prevalence of HBV infection. Interestingly, we found that HBsAg-positive DLBCL patients displayed less frequent involvement of the small and large intestine, which was not reported by previous studies.

In the era of DLBCL sub-classification, with cell-of-origin (COO) algorithms having entered clinical practice and being included in the 2016 revision of the WHO classification, we investigated the proportion of the non-GCB subtype in HBsAg-positive and -negative DLBCL patients, and the treatment response and outcome between the GCB and non-GCB subgroups in HBsAg-positive DLBCL patients. We identified 63% in the HBsAg-positive group as the non-GCB subtype, without a significant difference between the two groups, which suggests HBV infection might not participate in certain downstream molecular pathways, leading to GCB and non-GCB subgroup involvement. In addition, for HBsAg-positive DLBCL patients, the OS and remission rate did not differ between the GCB and non-GCB subtypes. Thus, our study did not find a significant representation of the COO subtype in HBsAg-positive DLBCL, which may have an influence on the prognosis. However, due to the small sample size and the lack of the gold standard COO classification by gene expression profile, further studies on the COO in HBsAg-positive patients are required.

MYC protein expression has been shown to be a poor prognostic factor in the rituximab era, which is compounded by BCL2 co-expression (double-positive or double-expressor lymphomas). Some study groups have reported that a high proliferation index (> 60–80%) is associated with poor prognosis. Several studies have shown positive immunoreactivity for p53 protein to be a poor prognostic indicator in DLBCL. To date, there have been no related reports in HBsAg-positive DLBCL patients. Our study showed that coexpression of BCL2 and MYC had a significantly worse outcome in HBsAg-positive DLBCL patients, while high expression of Ki-67 proliferation index, BCL2, MYC, and p53 did not affect prognosis in HBsAg-positive DLBCL patients. This result suggests that the OS of HBsAg-positive DLBCL patients could be improved by first-line chemotherapy combined with new targeted drugs such as ibrutinib.

Additionally, our study showed that there was a significant difference in the expression of MYC between the HBsAg-positive and -negative group. The expression rate of MYC in the HBsAg-positive group was significantly lower than that in the HBsAg-negative group. In contrast, Huang and colleagues [38] reported the reverse finding, showing that HBx expression was significantly associated with high-level expression of MYC. Liu and colleagues [26] found that the rate of MYC and BCL2 gene rearrangements in the HBV-infected group was significantly higher than that in the HBV-free group. It is likely that HBx antigen may contribute to the MYC signaling pathway and mediate lymphomagenesis in HBV+ patients. However, acording to Ren and colleagues’s report [36], the HBV-associated gene expression signature is impacted by the enrichment of genes regulated by BCL6, FOXO1, and ZFP36L1. So further multicenter clinical studies on larger cohorts of HBV-associated DLBCL are required for the identification and validation of candidate driver genes and will shed light on the complex mechanism underlying HBV infection and B-cell lymphomagenesis.

Most importantly, our study showed that there was no statistically significant difference in OS between HBsAg-positive and -negative patients. We also found that there was better OS in HBsAg-positive DLBCL patients treated with R-CHOP compared with those who received CHOP. Our results were inconsistent with the findings of most of previous studies [24–29]. We believe that there are two main reasons for this anomaly. On one hand, the poor prognostic factors between HBsAg-positive and -negative groups were similar except B symptoms. Furthermore, in our study, there was no difference in the use of rituximab and treatment response with R-CHOP regimen between HBsAg-positive and -negative DLBCL patients, which contributed to the similar prognosis of these two groups. On the other hand, although HBsAg-positive DLBCL patients had a higher risk of developing hepatic dysfunction and HBV reactivation during anti-tumor therapy, hepatic dysfunction higher than grade 2 and HBV reactivation both occurred in only 6.2% of HBsAg-positive DLBCL patients. Fatal hepatitis due to HBV reactivation in patients using rituximab has been reported. However, prophylactic antiviral therapy reduces the occurrence of potentially serious hepatic complications, allowing more patients to continue chemotherapy uninterrupted, and resulting in a trend towards improved OS. In our study, HBsAg-positive patients were routinely administered lamivudine or entecavir prophylactic treatment, starting from the beginning of chemotherapy and stopping one year after the completion of chemotherapy. Only two HBsAg-positive patients developed severe liver failure. Under antiviral prophylaxis, the survival of DLBCL patients with HBV infections was comparable to that of HBV-negative patients. A study by Wang et al. also showed that there was no statistically significant difference in OS between HBsAg-positive and -negative patients, while the OS of HBsAg-positive patients with hepatic dysfunction during chemotherapy was significantly shorter than that of patients without liver dysfunction [29], suggesting that hepatic dysfunction is a poor prognostic factor and contributes to poor outcome. The latest report by Huang et al. displayed that the mOS of HBV-negative and HBV carriers who received antiviral prophylaxis was similar. However, the mOS of HBV carriers who did not receive antiviral prophylaxis was significantly shorter than HBV carriers who received antiviral prophylaxis, indicating that antiviral prophylaxis improves OS in HBsAg-positive DLBCL patients [31].

## Conclusion

The current study showed that HBsAg-positive DLBCL has unique clinicopathological features and poor prognostic factors. HBsAg-positive DLBCL patients may benefit from prophylactic antiviral treatment and the use of rituximab significantly improved OS in HBsAg-positive DLBCL patients.

## Supplementary Information

**Additional file 1.** Clinicopathological and prognostic features of hepatitis B virus-associated diffuse large B-cell lymphoma: A single-center retrospective study in China.

## Data Availability

The data used to support the findings of this study are available from the corresponding author by request.
