# Supplementary material for: Clinicopathological and prognostic features of hepatitis B virus-associated diffuse large B-cell lymphoma: a single-center retrospective study in China
Source: Infect Agent Cancer. 2021 Aug 17;16:57. doi: 10.1186/s13027-021-00396-x (PMC8369744; doi:10.1186/s13027-021-00396-x)

**Additional file 1**

**Table S1. Treatment response in patients with R-CHOP regimen** **between HBsAg-negative and positive DLBCL groups**

|  | HBV+ | HBV- | P Value |
| --- | --- | --- | --- |
| Complete response | 42 | 114 | 0.105 |
| Partial response | 15 | 28 |  |
| Stable disease | 0 | 1 |  |
| Progressive disease | 3 | 0 |  |

**Table S2. Treatment response in patients with CHOP regimen between HBsAg-negative and positive DLBCL groups**

|  | HBV+ | HBV- | P Value |
| --- | --- | --- | --- |
| Complete response | 36 | 82 | 0.962 |
| Partial response | 25 | 55 |  |
| Stable disease | 1 | 3 |  |
| Progressive disease | 4 | 10 |  |

**Table S3. Multivariate Cox proportional hazards model for analysis of survival in HBsAg-negative DLBCL patients**

| Characteristics | P value | HR | 95% CI | |
| --- | --- | --- | --- | --- |
|  |  |  | Lower | Upper |
| Clinical staging | 0.014 |  |  |  |
| I/II |  | 1 | - | - |
| III/IV |  | 2.999 | 1.250 | 7.191 |
| Treated with rituximab | 0.006 |  |  |  |
| Yes |  | 0.318 | 0.140 | 0.724 |
| No |  | 1 | - | - |

**Figure S1.** **Survival analysis of HBsAg-negative and positive diffuse large B-cell lymphoma patients at different age groups**


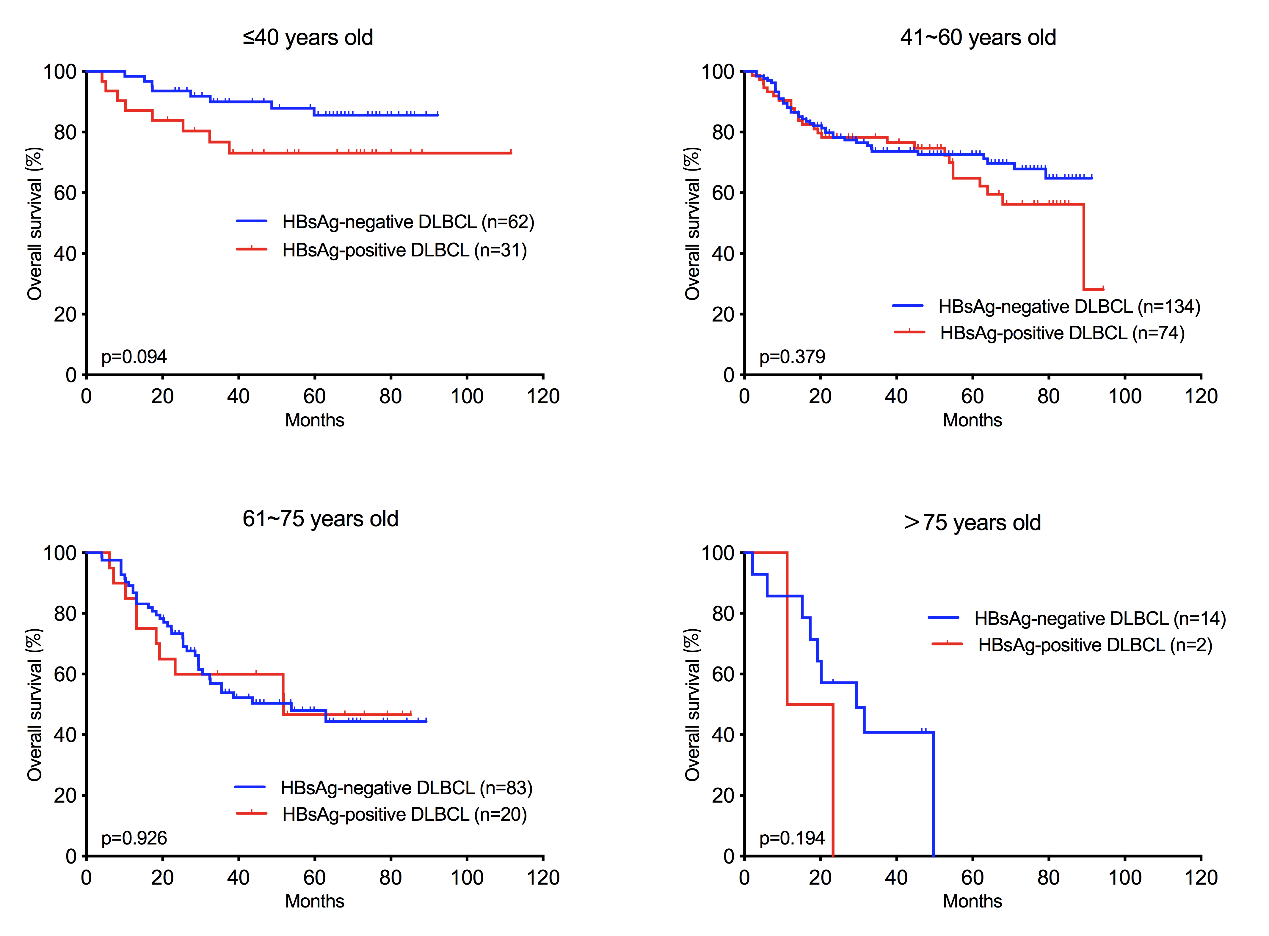


**Figure S2. Survival analysis of HBsAg-negative diffuse large B-cell lymphoma patients.** A. OS of HBsAg- negative DLBCL patients of more than 60 years old and less than 60 years old. B. OS of clinical staging in HBsAg- negative DLBCL patients. C. OS of HBsAg- negative DLBCL patients with and without elevated LDH. D. OS of HBsAg- negative DLBCL patients with and without spleen involvement. E. OS of HBsAg- negative DLBCL patients with and without B symptoms. F. OS of HBsAg- negative DLBCL patients treated with R-CHOP or CHOP regimens. G. OS of HBsAg- negative DLBCL patients with and without coexpression of BCL2 and MYC. H. OS of HBsAg- negative DLBCL patients between GCB subgroup and non-GCB subgroup.


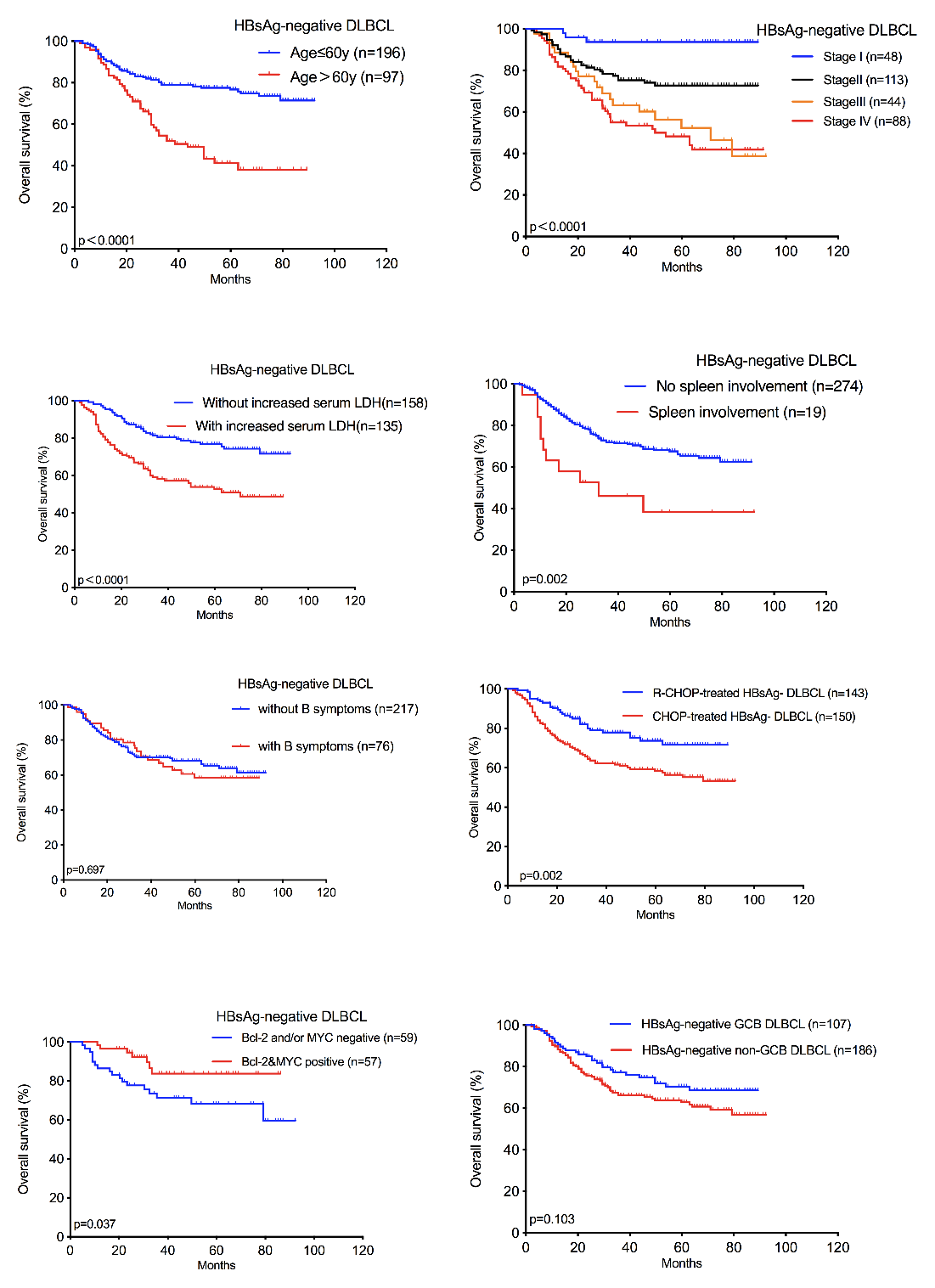

Supplement: Supplementary file 1 — Additional file 1. Clinicopathological and prognostic features of hepatitis B virus-associated diffuse large B-cell lymphoma: A single-center retrospective study in China. [file 13027_2021_396_MOESM1_ESM.docx]
